# The complete chloroplast genome sequence of a vulnerable legume species *Dalbergia tonkinensis* prain in Vietnam

**DOI:** 10.1080/23802359.2019.1641441

**Published:** 2019-07-17

**Authors:** Changyoung Lee, Yong-In Kim, Soo-Yong Kim, Tran The Bach, Sangmi Eum, Nguyen Trung Thanh, Sangho Choi

**Affiliations:** a International Biological Material Research Center, Korea Research Institute of Bioscience and Biotechnology, Daejeon, Republic of Korea;; b Department of Botany, VNU University of Science, Hanoi, Vietnam;; c Multidisciplinary Genome Institute, Life Science Hall, Hallym University, Chuncheon, Republic of Korea;; d Institute of Ecology and Biological Resources (IEBR), Vietnam Academy of Science and Technology (VAST), Hanoi, Vietnam

**Keywords:** *Dalbergia tonkinensis*, vulnerable species, chloroplast genome, phylogenetic analysis

## Abstract

*Dalbergia tonkinensis* is a critically vulnerable tree species that is distributed in Vietnam and Hainan Island of China. The complete chloroplast genome sequence of *D. tonkinensis* was characterized using Illumina pair-end sequencing. The cpDNA is 156,086 bp in length and contains a pair of 25,720 bp inverted repeats, one large single copy region of 85,761 bp, and one small single copy region of 18,885 bp. It contains 131 genes including 86 protein-coding genes, 36 tRNAs, eight rRNAs, and one pseudogene. The overall G + C content of the whole genome is 36.1%. Phylogenetic analysis based on 35 chloroplast genomes of Papilionoideae including *Cercis glabra* (as an outgroup) indicates that all the species of the Dalbergioids *sensu lato* formed a monophyletic clade and *D. tonkinensis* formed a sister relationship with the *D. hainanensis* and *D. odorifera* group.

*Dalbergia tonkinensis* is a 5–13 m tall floral tree species belonging to Papilionoideae de Candolle (Fabaceae) and is distributed in Vietnam and Hainan Island of China (Nguyen et al. 2018). This plant was encountered the serious threat as the results of human activities and overexploitation for economic uses. It has been classified as a critically vulnerable plant listed in the Vietnam Red List (Dang and Nguyen 2007) and IUCN Red list (Ban 1998). In this study, the complete chloroplast genome of *D. tonkinensis* was reconstructed based on Illumina sequencing to provide the underlying information for genetic and conservation studies and explore possible phylogenetic relationship within Papilionoideae.

The plant material was sampled at Bac Huong Hoa nature reserve (Vietnam). The voucher specimen (KRIB0077649) was deposited in herbarium of KRIB. The total genomic DNA was sequenced using the Illumina Hiseq 2500 platform. The pair-end library was comprised 43,285,230 reads (length 2 × 101 bp). The Geneious *de novo* assembler was used to assemble the trimmed paired-read set. generated 565,298 contigs. The draft chloroplast genome of 156,086 bp achieved by identifying the repetitive inverted repeats sequences at 131,558 bp contig suspected of chloroplast genome with a mean coverage of 287.8 X. The gene annotations were conducted by using Geneious v 11.1.5 (Biomatters Ltd., New Zealand) via comparison with chloroplast genomes of *D. odorifera* T. Chen (MF668133) and *D. hainanensis* Merr. et Chun (MF926268). The annotated chloroplast genome sequence was submitted to the GenBank (MK599470).

The complete chloroplast genome of *D. tonkinensis* has a total length of 156,086 bp, with a pair of inverted repeats of 25,720 bp that separate a large single copy region of 85,761 bp and a small single copy region of 18,885 bp. The chloroplast genome contained 131 genes including 86 protein-coding genes, 36 tRNA genes and 8 ribosomal RNA genes. Among these genes, 15 genes (*atp*F, *ndh*A, *ndh*B, *pet*B, *pet*D, *rpl*2, *rpl*16, *rpo*C1, *rps*12, *ycf*68, *trn*A-UGC, *trn*I-GAU, *trn*K-UUU, *trn*L-UAA, *trn*V-UAC) have one intron and three genes (*clp*P, *rps*12, *ycf*3) have two introns. The *ycf*1 gene was inferred to be pseudogenes. Most of the genes occurred as a single copy; however, six protein-coding genes (*ndh*B, *rpl*2, *rpl*23, *rps*7, *rps*12, *ycf*2), seven tRNA genes (*trn*A-UGC, *trn*I-CAU, *trn*I-GAU, *trn*L-CAA, *trn*N-GUU, *trn*R-ACG, *trn*V-GAC) and four rRNA genes (*rrn*16, *rrn*23, *rrn*4.5, *rrn*5) in the IR regions were duplicated. The overall G + C content of chloroplast genome is 36.1%,. respectively

Phylogenetic analysis was performed using chloroplast coding sequences of *D. tonkinensis* and those of 35 related species of Papilionoideae including *Cercis glabra* Pamp, as an outgroup. These chloroplast genomes were aligned by using MAFFT (Katoh and Standley 2013). Maximum-likelihood tree was generated using IQ-TREE (Nguyen et al. 2015) based on TVM + F +R3 model with ML + ultrafast bootstrap (Minh et al. 2013) 1000 replicates. As shown in the highly resolved ML phylogenetic tree (Figure 1), all the species of the Dalbergioids *sensu lato* formed a monophyletic clade and *D. tonkinensis* formed a sister relationship with the *D. hainanensis* and *D. odorifera* group.

**Figure 1.**
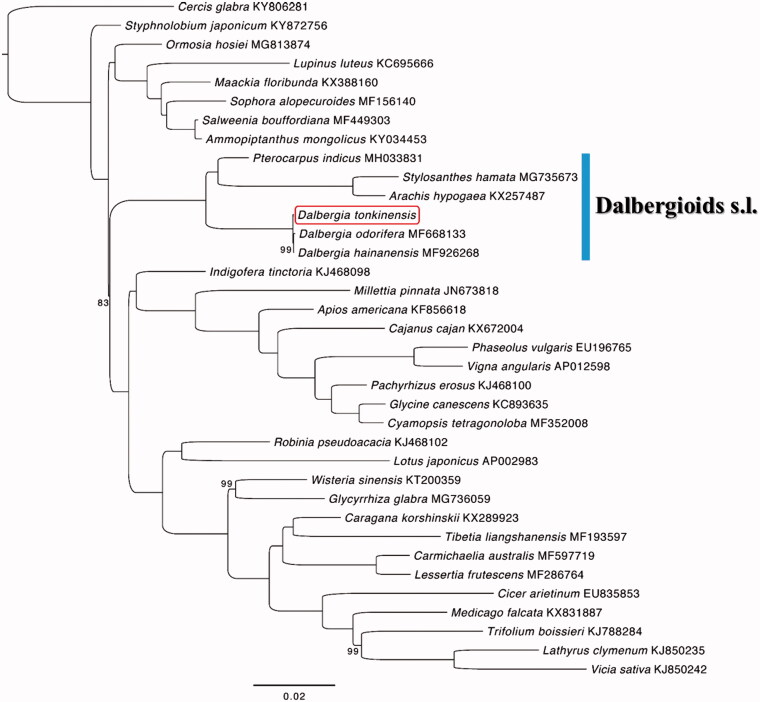
Maximum-likelihood phylogenetic tree based on 35 complete chloroplast genomes of Papilionoideae including *Cercis glabra,* as an outgroup. The number on each node indicates the bootstrap value less than 100. The position of *Dalbergia tonkinensis* is shown in a red blank box.
